# Different Levels of DNA Methylation Detected in Human Sperms after Morphological Selection Using High Magnification Microscopy

**DOI:** 10.1155/2016/6372171

**Published:** 2016-04-11

**Authors:** Nino Guy Cassuto, Debbie Montjean, Jean-Pierre Siffroi, Dominique Bouret, Flora Marzouk, Henri Copin, Moncef Benkhalifa

**Affiliations:** ^1^ART Unit, Drouot Laboratory, Drouot Street 21, 75009 Paris, France; ^2^Reproductive Medicine and Biology Service, Saint-Joseph Hospital, 26 Boulevard de Louvain, 13008 Marseille, France; ^3^Medical Genetics and Embryology Department, Armand-Trousseau Hospital (AP-HP), 28 Avenue du Dr. Arnold Netter, 75012 Paris, France; ^4^Reproductive Biology & Medical Cytogenetic Laboratory, University Hospital & School of Medicine, Picardy University Jules Verne, 80025 Amiens, France

## Abstract

*Objective*. To analyze DNA methylation levels between two groups of spermatozoa taken from the same sample, following morphological selection by high magnification (HM) at 6100x microscopy. A prospective study was conducted and studied 876 spermatozoa from 10 randomly selected men. Sperm morphology was characterized at HM according to criteria previously established. High-scoring Score 6 and low-scoring Score 0 sperm were selected. Sperm DNA methylation level was assessed using an immunoassay method targeting 5-methylcytosine residues by fluorescence microscopy with imaging analysis system to detect DNA methylation in single spermatozoon.* Results*. In total, 448 S6 spermatozoa and 428 S0 spermatozoa were analyzed. A strong relationship was found between sperm DNA methylation levels and sperm morphology observed at HM. Sperm DNA methylation level in the S6 group was significantly lower compared with that in the S0 group (*p* < 10^−6^), OR = 2.4; and *p* < 0.001, as determined using the Wilcoxon test.* Conclusion*. Differences in DNA methylation levels are associated with sperm morphology variations as observed at HM, which allows spermatozoa with abnormal levels to be discarded and ultimately decrease birth defects, malformations, and epigenetic diseases that may be transmitted from sperm to offspring in ICSI.

## 1. Introduction

In reproductive physiology, many abnormalities may occur during spermatogenesis, resulting in spermatozoa defects. Such defects include those in the sperm morphology, numerical or structural chromosomal abnormalities, abnormal chromatin, and sperm DNA defects, which lead to a poor fertilization rate, chaotic early embryonic development, high rates of miscarriage, or birth defects [1]. Specifically, spermatozoa can undergo DNA damage through a number of processes, including abortive apoptosis, oxidative stress, associated genital tract infection, defects in spermiogenesis, mild scrotal heating, and environmental and physical factors such as radiation or chemical exposure [1]. Today, the investigation of male infertility is largely limited to clinical examination, ultrasound, and assessment of hormonal, karyotype, and sperm parameters. Assessing sperm DNA will likely provide invaluable information regarding sperm quality to further understand male infertility. In gametes DNA methylation is a major factor controlling imprinted gene expression during embryo development. Altered methylation profiles in gametes can have negative effects on offspring.

Recent studies have reported that epigenetic modifications in mature spermatozoa play an important role in the developing embryo; alterations in epigenetic patterns may increase the risk of fertilization failure, dysfunction of embryogenesis, preterm birth, low birth weight, congenital anomalies, and perinatal mortality [2]. DNA methylation, an important epigenetic marker, increases with age [3–5] and is also altered in oligozoospermia sperms [6], potentially contributing to fertility impairment in couples with unexplained infertility [7]. Additionally, studies have identified associations between altered paternal sperm DNA methylation and the risk of neurological diseases [8], as well as autism in offspring [9].

During early embryogenesis, the spermatozoon delivers a novel epigenetic signature to the egg, which is a crucial step in normal embryonic development [10]. Findings reported in human assisted reproductive technologies (ART) have confirmed that DNA methylation errors are more prevalent in patients with oligozoospermia. These methylation errors are subsequently transmitted to the embryo, conferring a potential risk of imprinting disorders to the offspring [11]. Such epigenetic aberrations have detrimental consequences not only in early embryonic development but also ultimately in the fate of the fetus [12].

Previously, we reported a decreased risk of major birth defects in children of couples in which high magnification (HM) microscopic sperm observation and selection before injection were used to discard low quality or “Score 0” spermatozoa for intracytoplasmic morphologically selected spermatozoa injection (IMSI). Score 0 (S0) was defined by a nuclear-shape disorder with an abnormal base and/or a nuclear asymmetrical extrusion and/or invagination of the nuclear membrane and at least one large vacuole [13].

Although sperm quality and DNA integrity may have predictive value in early embryonic development and pregnancy rate in ART, technical reasons unfortunately prevent this assessment prior to intracytoplasmic injection. This study was aimed at assessing the relationship between sperm head morphology and DNA methylation levels.

## 2. Materials and Methods 

### 2.1. Study Population

This study evaluated spermatozoa from a population of 10 men without male infertility, randomly selected from couples undergoing assisted reproductive technologies (ART) for female infertility. All treatments concerned only the women; 4 women were treated for tubal infertility, 4 were treated for unexplained infertility, 1 was treated for anovulation, and 1 was treated for poor response.

The prospective design of this study was approved by the local ethical committee of Bluets Hospital and conducted at the Assisted Reproduction Unit of the Drouot Laboratory (2015 January 26). Institutional Review Board (IRB) approval was not mandatory because the study design was noninterventional. Patients signed a consent form informing them that their semen would be observed under high magnification and that sperm DNA tests would be carried out.

### 2.2. Data Collection

Ejaculates were collected in sterile containers by masturbation after 2 or 3 days of sexual abstinence. Only fresh ejaculates were used for the study; epididymal, testicular, and cryopreserved sperm samples were not included. The 10 ejaculated samples were liquefied for 15 minutes at room temperature prior to analysis. In a previous study, we reported a scoring scale from 6 to 0 and described a new classification of sperm morphology at high magnification in real time [14]. In the present study, sperm parameters from the 10 men were analyzed according to the World Health Organization guidelines [15]; and we report also the percentage of the sperm heads given Score 6 (S6) and Score 0 (S0) from the sperm gradient preparation (Table 1).

### 2.3. Sperm Preparation

Sperm migration was performed with a bilayer discontinuous gradient. In a conical tube, 1 mL liquefied semen sample was layered on two-layer concentration gradients containing 45% and 90% of Isolate Sperm Separation Medium (Cat. N° 99264; Irvine Scientific, Santa Ana, CA, USA). The tube was centrifuged at 300 ×g for 15 min. The supernatant was discarded and the sperm pellet was washed with Ham's Medium (Cat. N° 99168; Irvine Scientific) containing 5% human serum albumin (HSA; Cat. N° 9988; Irvine Scientific) and then centrifuged at 300 ×g for 5 min. The final pellet containing total migrated spermatozoa was resuspended in 300 *μ*L medium.

### 2.4. Sperm Morphology Assessment and Sperm Selection

Each freshly washed pellet was placed in a glass-bottom culture dish (GWST-5040; WillCo Wells B.V., Amsterdam, Netherlands) under light mineral oil (Cat. N° 9305; Irvine Scientific).

High magnification selection was performed under an inverted microscope (Leica DMI 3000 Leica Microsystems, France) equipped with Nomarski contrast optics. High-power magnification was achieved using a polarization light with a magnification of 1500x and a zoom of 6100x.

**Figure 1:**
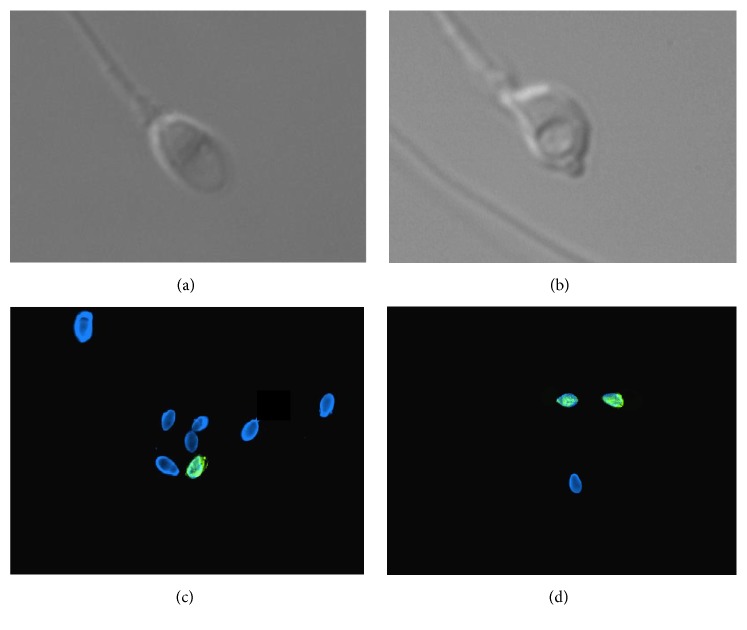
Spermatozoa classification and 5-methylcytosine immune labeling in S0 and S6 spermatozoa (a): “high-scoring” spermatozoon Score 6 at high magnification (6100x). (b): “low-scoring” spermatozoon Score 0 at high magnification (6100x). Cassuto Barak Classification for motile sperm cells. Fertil Steril 2009. (c): 5-methylcytosine detection in S6 spermatozoa (100x). Merge Dapi (Blue) and FITC (Green). Spermatozoa showing green labeling display detectable level of DNA methylation. (d): 5-methylcytosine detection in S6 spermatozoa (100x). Merge Dapi (Blue) and FITC (Green). Spermatozoa showing green labeling display detectable level of DNA methylation.

All motile spermatozoa were examined and observed in three dimensions in a micropipette at high magnification (6100x). Spermatozoa morphology was characterized by the head shape, the presence of a vacuole in the nucleus, and the head base. The formula for the scoring system is as follows: two points for a normal head, three points for a head without a vacuole, and one point for a normal base. High-scoring morphology spermatozoa were given S6 (Figure 1(a)). Low-scoring (S0) spermatozoa were defined by a nuclear-shape disorder with an abnormal base and/or a nuclear asymmetrical extrusion and/or invagination of the nuclear membrane and at least one large vacuole (Figure 1(b)). None of the factors used to define abnormal morphology of the sperm nucleus are visible at low magnification.

For each patient, spermatozoa given S6 and S0 were selected and extracted separately from the same pellet. We then prepared 2 glass slides: one with a spot containing 50 S6 spermatozoa and the other with a spot containing 50 S0 spermatozoa in PBS. The slides were air-dried and stored at −20°C.

### 2.5. Sperm DNA Methylation Level Assessment

To investigate sperm DNA methylation, we used a modified immunoassay using the antibody sandwich method with a second antibody labeled with FITC adapted from Benchaib [16, 17]. The immunoassay was analyzed with a fluorescent microscope.

Briefly, after sperm permeabilization and decondensation to facilitate access of the antibodies to the 5-methylcytosine (5-mC) DNA base, we simultaneously and systematically fixed all selected S0 and S6 spermatozoa with an acetone solution on a glass slide followed with a 4% par formaldehyde solution. After dehydration, slides were incubated with a mouse antibody specific to 5-mC (Cat N° ab 73938, Abcam, UK) for 1 hour at room temperature for hybridization. The slides were then washed to eliminate excess antibody. For the sandwich method, a second antibody labeled with FITC was used. Slides were incubated with goat anti-mouse antibody (Cat N° ab 97229, Abcam, UK) for 1 hour at room temperature and then washed 3 times to eliminate nonspecific hybridization. Spermatozoa incubated with buffer instead of the primary antibody were used as a negative control. The slides were dehydrated in a series of ethanol solutions and counterstained with 4,6-diamino-2-phenylindole (DAPI) prior to analysis with a fluorescent microscope. To maximize the efficiency of the immunoassay sandwich method, we reduced the fluorescent noise of the second antibody. In addition, we confirmed that, without adding the first antibody, the second antibody did not yield significant signal from sperm DNA hybridization beyond significant background. Slides were stored at 4°C in a dark chamber until visualization of sperm head immunofluorescence with an epifluorescence microscope.

After DNA staining and image acquisition using a DAPI filter, DNA stained in blue, we switched to a FITC filter and assessed sperm DNA methylation stained in green. As an internal control, we used 16 selected migrated sperm samples from patients with normal sperm parameters according to WHO 2010 criteria [15] (Table 2).

Quantitative analysis was performed using an imaging system from Applied Imaging Leica (Leica Microsystems, France) for fluorescent sperm DNA analysis to reflect the levels of 5-mC DNA bases. To define the average percentage of 5-mC in normal sperms, we analyzed 200 spermatozoa from the total pellet migrated from 16 men with normal sperm parameters by counting the number of spermatozoa with a head totally stained green by 2 different observers. Methylation levels varied from 4% to 22% with an average of 13% for the normal sperm controls. Based on this analysis, we considered the cutoff for a normal methylation profile as having less than 13% methylated DNA in sperms (Table 3).

This technique revealed spermatozoa with levels of methyl cytosine that can be detected with antibodies and visualized as green by fluorescence microscopy with magnification 100x using the sandwich method. It is possible that this immunohistochemistry method fails to detect spermatozoa at lower methylation levels than those observed.

### 2.6. Statistical Analysis

Statistical analyses were performed using R version 2.10.1 [18]. The R glm function, followed by the R step function, was applied to the data to obtain significant values based on the Akaike information criterion. Values were considered statistically significant with *p* values < 0.05. Differences in continuous data are presented as a mean difference and 95% confidence interval (CI). Differences in categorical data were expressed in terms of odds ratios (OR) and 95% CI. When necessary, data were compared using nonparametric tests, such as the Wilcoxon test used to compare matched series for few samples.

## 3. Results

In total, 448 S6 spermatozoa and 428 S0 spermatozoa were identified and analyzed; we report the percentage of methylated spermatozoa. 5-methylcytosine immunolabeling pattern in S6 spermatozoa and S0 spermatozoa is depicted in Figures 1(c) and 1(d), respectively.

**Figure 2:**
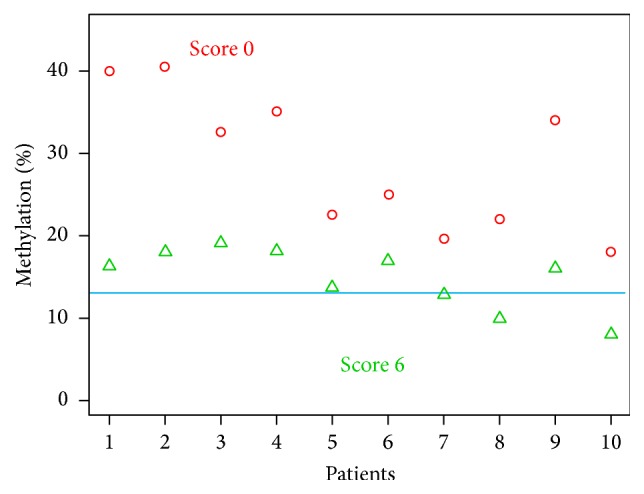
Percentage of methylated sperm for Score 0 and Score 6 for each patient analyzed (cutoff 13%).

As shown in Figure 2, S0 spermatozoa had DNA methylation levels significantly higher than the 13% cutoff. The average DNA methylation was 14.9% versus 28.9% for S6 and S0, respectively.

The medians 16.2% (SD 4%) from 8% to 19.1% versus 28.8% (SD 8%) from 18% to 40.5% were found for S6 and S0, respectively (Figure 2).

**Figure 3:**
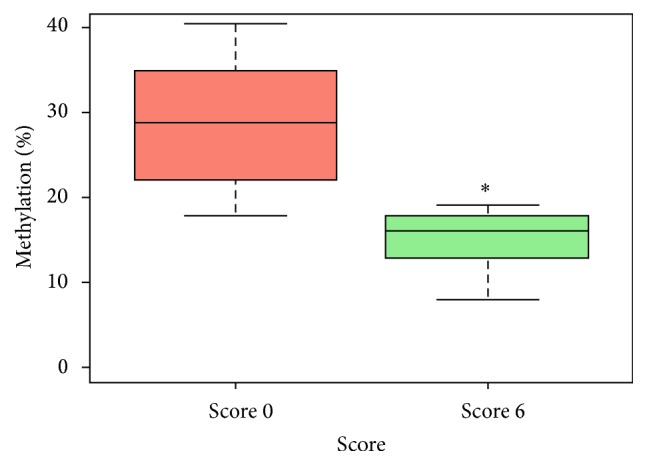
The overall sperm DNA methylation patterns from the 10 patients according to Score 6 and Score 0. ^*∗*^ *p* < 0.001.

Overall, S6 spermatozoa (*n* = 448) showed significantly lower methylation level (66 labelled spermatozoa over 448 analyzed) than S0 spermatozoa (122 out of 428) (*χ* ^2^ = 23.82; *p* < 10^−6^); OR = 2.4 (95% CI 1.33–1.55). Wilcoxon signed rank test was used to examine the significance of the ratio of DNA methylation between S6 and S0 in matched patients. We identified a strong relationship between 5-mC DNA methylation ratio and the spermatozoa morphology at HM, regardless of the distribution of the variables (*p* < 0.001) (Figure 3).

## 4. Discussion

In this preliminary study, to the best of our knowledge, we demonstrate for the first time a distinct relationship between sperm head morphology and DNA methylation levels. We investigated DNA methylation in spermatozoa by immunostaining for 5-mC. Thus far, DNA methylation has been evaluated either at specific loci [6, 11, 19] or on the genome-wide scale [20] on a nonspecific population of spermatozoa, but not on selected populations of spermatozoa. The novelty of this present study in evaluating genome-wide DNA methylation lies in the determination of the ratio of DNA methylation in sperms having two different morphologies in the same sample using high magnification microscopy.

Cytosine methylation is stably distributed across the genome; this comparatively static landscape is in marked contrast with that of the genome during the events of fertilization, during which methylation of the paternal genome is globally reprogrammed. After fertilization, global methylation reprogramming results in more highly methylated DNA in spermatozoa than in eggs [21]. Until now, the mechanisms regulating spermiogenesis remain unclear and appear to permit de novo genetic polymorphisms transmittable to the next generation [22]. By analyzing sperm DNA methylation and RNA transcripts in spermatozoa, a recent study showed the paternal contribution and the crucial role of sperm epigenetic in embryonic development [23].

Indeed, DNA methylation is a key regulator of transcription and contributes to gene expression defects observed in men with poor semen parameters, low sperm counts, and low motility [24]. In men with impaired spermatogenesis, the sperm epigenetic landscape is frequently altered. However, whether the sperms of infertile men with abnormal semen parameters exhibit relatively hyper- or hypo-DNA methylation remains subject to ongoing debates. Moreover, the exact mechanism responsible for sperm DNA methylation defects in infertile men remains unknown. Genome-wide analysis of sperm from abnormal semen samples revealed global sperm DNA hypermethylation [20].

Several studies have reported that sperm DNA methylation errors are more frequent in men with abnormal semen criteria than in normozoospermic sperm [6, 19, 25]. More recently, it has been reported that defective and apoptotic sperm may be associated with hypermethylated sperm nuclear DNA [26]. Epigenetic defects, primarily aberrant methylation of certain genes, can alter spermiogenesis, resulting in poor sperm parameters such as concentration or morphology. These defects can generate defective spermatozoa with increased risk of adverse effects on embryo development, imprinting disorders, and health implications for the next generation [27].

Many repeated failures in ICSI are often connected with defects in the injected spermatozoa [28]. In a previous study, by deselecting abnormal spermatozoa before ICSI through highly magnified sperm observation in real time permitted to discard S0 spermatozoa, we observed decreased birth defects of the offspring of couples in the IMSI program [13]. In a previous study, we demonstrated that S0 spermatozoa are associated with poor prognosis for embryonic development. ICSI of an S0 spermatozoon compared to an S6 had negative impacts on early embryonic development and did not reach an expanded blastocyst stage on day 5 [14]. The factors contributing to defective embryonic development with S0 spermatozoa are not related to spermatozoa chromosomal status [29] or DNA fragmentation level but are related to high levels of sperm chromatin decondensation [30], which may be deleterious for early embryonic development and outcome. These observations suggest that S0 spermatozoa are affected at least during the maturation stage of spermiogenesis. In the present study, we have investigated the extent to which S0 spermatozoa with poor morphology display different methylation profiles from S6 spermatozoa.

During early development, methylation that occurs too early may generate an aberrant DNA methylation pattern, subsequently switching off gene expression, resulting in protein deficiency during sperm maturation, or the early embryo development. These data identify variation in methylation in each individual sperm, suggesting that each spermatozoon may carry similar or/and more subtle alterations. Further studies, however, are required to determine the relationship between sperm DNA hypermethylation and phenotype and disease risk in selected populations.

The technique used for DNA methylation testing is invasive, as it requires fixing and staining spermatozoa, which causes irreversible damage and excludes the possibility for use in oocyte fertilization. Sperm examination in real time at high magnification may be a preferable, noninvasive alternative to DNA methylation assessment by standard methods. Based on our results, identifying sperm morphology may effectively predict sperm DNA methylation profiles. This process will enable us to discard spermatozoa with a higher risk of methylation aberration due to a predictably poor prognosis and ensure safer biological and clinical outcomes for ICSI.

## 5. Conclusions

These data reveal a clear significant correlation between sperm head morphology and DNA methylation profile. High magnification visualization of sperms in real time provides the opportunity to identify and discard low quality spermatozoa, which have a higher risk of DNA hypermethylation, prior to injection, ultimately improving ART outcomes by decreasing the risk of birth defects, major malformations, and epigenetic diseases in the offspring through ICSI.

## Figures and Tables

**Table 1:** Sperm parameters and percentage of Score 6 and Score 0 from the 10 patients.

Patients	Age	BMI	Concentration (millions/mL)	Total motility (%)	Progressive motility (%)	Morphology (%)	Score 6/Score 0 (%)	ART
P1	45	22	60	50	30	10	5/30	IMSI
P2	39	23	100	50	40	12	10/40	IMSI
P3	50	23	9	40	30	5	5/50	IMSI
P4	37	23	70	55	40	16	5/45	IMSI
P5	47	30	57	60	50	5	15/30	IVF
P6	37	22	55	20	10	11	10/40	IMSI
P7	45	22	37	50	40	7	5/35	IMSI
P8	45	23	62	50	40	11	10/35	IUI
P9	46	25	30	60	50	10	5/45	IVF
P10	37	22	82	50	40	7	5/50	IUI

P: patients;  BMI: Body Mass Index.

**Table 2:** The sperm parameters for the 16 fertile men included as control group.

Patients controls	Concentration millions/mL	Motility (%)	Vitality (%)	Morphology (%)
PC1	103	60	84	14
PC2	15.2	45	76	12
PC3	112	55	88	9
PC4	78	60	85	11
PC5	140	70	84	9
PC6	17	40	77	7
PC7	20	50	78	12
PC8	42	45	79	7
PC9	61	40	73	10
PC10	62	47	86	10
PC11	32	55	87	12
PC12	50	57	81	14
PC13	18	49	83	10
PC14	48	54	83	11
PC15	109	45	88	16
PC16	61	57	78	15

PC: patient control.

**Table 3:** Percentage of methylated sperm in the total migrated sperm for the 16 men control given by 2 different observers.

Patients controls	First observer (%)	Second observer (%)
PC1	7	8
PC2	3	5
PC3	7	8
PC4	14	17
PC5	19	15
PC6	18	20
PC7	9	10
PC8	22	18
PC9	17	16
PC10	5	9
PC11	20	24
PC12	16	12
PC13	6	5
PC14	8	7
PC15	9	12
PC16	24	19
Average	12.7	12.8

PC: patient control.
